# External validation of clinical risk prediction score for elderly treated with endovascular thrombectomy

**DOI:** 10.1007/s00415-024-12535-6

**Published:** 2024-07-02

**Authors:** Brian Anthony B. Enriquez, Thor Håkon Skattør, Nicolaj Grønbæk Laugesen, Thomas Truelsen, Christian Georg Lund, Terje Nome, Mona K. Beyer, Mona Skjelland, Anne Hege Aamodt

**Affiliations:** 1 Department of Neurology, Oslo University Hospital, Rikshospitalet, Oslo, Norway; 2 Division of Radiology and Nuclear Medicine, Oslo University Hospital, Rikshospitalet, Oslo, Norway; 3 Department of Neurology, Stroke Center Rigshospitalet, Copenhagen, Denmark; 4 Faculty of Health and Medical Sciences, The University of Copenhagen, Copenhagen, Denmark; 5 Institute of Clinical Medicine, University of Oslo, Oslo, Norway; 6 Department of Neuromedicine and Movement Science, The Norwegian University of Science and Technology, Trondheim, Norway

**Keywords:** Thrombectomy, Elderly, Ischemic stroke, Prediction

## Abstract

**Background and aim:**

The thrombectomy in the elderly prediction score (TERPS) for functional outcome after anterior circulation endovascular therapy (EVT) in patients ≥ 80 years was recently developed. The aim of this study was to assess predictors of functional outcome in the elderly and validate the prediction model.

**Methods:**

Consecutive patients treated with EVT from the Oslo Acute Reperfusion Stroke Study were evaluated for inclusion. Clinical and radiological parameters were used to calculate the TERPS, and functional outcome were assessed at 3-month follow-up.

**Results:**

Out of 1028 patients who underwent EVT for acute ischemic stroke from January 2017 to July 2022, 218 (21.2%) patients ≥ 80 years with anterior ischemic stroke were included. Fair outcome, defined as modified Rankin scale ≤ 3 (mRS), was achieved in 117 (53.7%). In bivariate analyses, male sex (*p* 0.035), age (*p* 0.025), baseline National Institute of Health Stroke Scale (NIHSS, *p* < 0.001), pre-stroke mRS (*p* 0.002) and Alberta Stroke Program Early Computed Tomography score (ASPECTS, *p* 0.001) were associated with fair outcome. Significant predictors for fair outcome in regression analyses were lower pre-stroke mRS, adjusted odd ratio, (aOR) 0.67 (95% CI 0.50–0.91, *p* 0.01), NIHSS, aOR 0.92 (95% CI 0.87–0.97, *p* 0.002), and higher ASPECTS, aOR 1.22 (95% CI 1.03–1.44, *p* 0.023). The area under the curve (AUC) using TERPS was 0.74 (95% CI 0.67–0.80).

**Conclusions:**

The risk prediction score TERPS showed moderate performance in this external validation. Other variables may still be included to improve the model and validation using other cohorts is recommended.

**Trial registration:**

NCT06220981.

## Introduction

In clinical practice, patients above 80 years of age represent an increasing proportion of stroke patients due to the aging population. Endovascular therapy (EVT), a highly effective treatment for acute ischemic stroke (AIS) with proximal vessel occlusions, presently has no age limit. Although most patients in this age group may have considerable functional limitations as well as chronic comorbidities, the benefit of EVT in a proportion of patients with disabilities has been reported [1, 2].

The evidence of EVT in patients ≥ 80 years is limited as they have either been excluded or underrepresented in previous trials. In the HERMES study, only 198 of the 1287 (15.4%) study participants were ≥ 80 years, of which 91 (7%) belonged to the intervention group [3]. Analyses of elderly patients ≥ 80 years showed a reduced risk of death and benefit of EVT, although age remained a strong predictor of outcome. Systematic reviews and meta-analyses have shown that higher rates of morbidity and mortality, and lower chances of recanalization, are more common in the elderly [4, 5].

With inadequate representation in the randomized control trials and with worse technical and clinical outcomes in several studies, predictors of clinical outcome in the elderly could potentially be useful in selecting patient eligibility for EVT. Age, baseline National Institute of Heath Stroke Scale (NIHSS), and Alberta Stroke Program Early CT Score (ASPECTS) have been demonstrated as predictors of functional outcome after stroke and EVT [6–11]. The use of pre-stroke modified Rankin Scale (mRS) in assessing premorbid function in EVT patients is also gaining grounds for clinical use [12–16]. Thus, a tool that can aid clinicians in determining the elderly patient who is most likely to benefit from EVT is feasible.

Recently, a clinical risk prediction score—the thrombectomy in the elderly risk prediction score (TERPS)—to predict functional outcome of AIS patients after anterior circulation EVT in patients ≥ 80 years of age was developed [17]. The aim of this study was to assess predictors for functional outcome and validate the prediction model using an elderly subset of patients included in the Oslo Acute Reperfusion Stroke Study (OSCAR).

## Methods

The TERPS, a model derived from data on patients ≥ 80 years treated with EVT, is a simplified scoring system based on age, pre-stroke mRS, NIHSS and ASPECTS to predict functional outcome at 3-month follow-up [17]. TERPS score ranges from 0 to 18, wherein the probability of poor outcome with a score of 0 is estimated at 19%, while a full score probability of poor outcome is 98% (Table 1). At a cutoff value of seven points, patients below this score had better odds of achieving a fair outcome [17].

**Table 1:** Thrombectomy in the Elderly Risk Prediction Score (TERPS), minimum score 0, maximum score 18

Variable	Assigned score
Age	
80–84	0
85–89	1
> 89	3
Admission NIHSS	
0–12	0
13–17	2
18–21	4
> 21	5
ASPECTS	
9–10	0
7–8	1
< 7	2
Pre-stroke mRS	
0	0
1	3
2	5
> 2	8

*NIHSS* National Institutes of Health Stroke Scale, *ASPECTS* Alberta stroke program early CT score, *MRS* modified Rankin scale

**Fig. 1:**
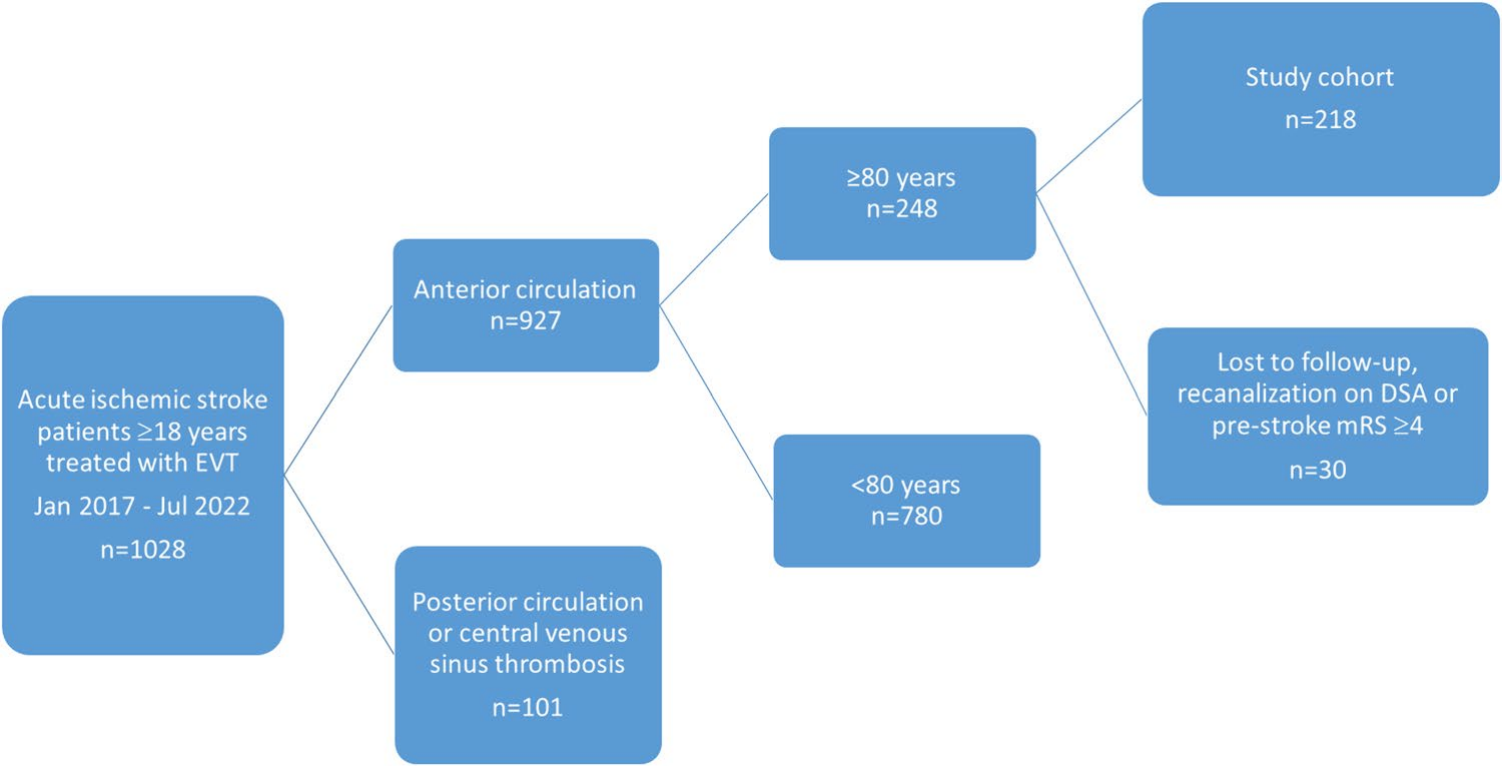
Flowchart for patient selection. EVT = endovascular therapy. DSA = digital subtraction angiography

Consecutive patients treated with EVT for acute ischemic stroke in the period between January 1st, 2017 and July 31st, 2022, included in the OSCAR study, were reviewed for inclusion in this study. The OSCAR study included all patients receiving acute endovascular reperfusion therapy in both anterior and posterior circulation, including both large vessel occlusion (LVO), medium vessel occlusion (MeVO) and central venous sinus thrombosis as approved by the regional ethics committee. Oslo University Hospital, Norway, is a comprehensive stroke center, receiving patients from primary stroke centers for EVT assessments. Stroke patients were initially screened with non-contrast head CT and CT angiography (CTA), with or without perfusion at the referring hospitals. Patients eligible for intravenous thrombolysis received treatment at the primary stroke center and were transferred to our center if they were possible candidates for EVT. Patients were considered for treatment if they had a pre-stroke mRS of up to 4 and a NIHSS score of ≥ 6. Patients with a NIHSS score of 3–5 were also considered if they had severe symptoms, like aphasia, and/or perfusion imaging showed significant penumbra. The pre-stroke mRS was initially assessed by the stroke neurologist upon admission, and later verified or adjusted by a mRS certified neurologist (BABE) as more patient information became available. In cases where there is discrepancy in the pre-Stroke mRS, the updated pre-stroke mRS was used in the analyses. For cases lacking pre-stroke mRS, retrospectively scoring was done using hospital records by the same mRS certified neurologist. A pre-stroke mRS of 1 was assigned to patients with stroke or stroke-like sequelae without resulting in functional disability, while a score of 2 was given to patients unable to perform previous activities they could perform before, expected of their age, due to various causes. Patients with posterior circulation stroke, recanalization seen on digital subtraction angiography before thrombectomy, patients lost to follow-up, and those with a pre-stroke mRS ≥ 4 were excluded from this study (Fig. 1).

Endovascular therapy was done up to 24 h from symptom onset with guidance of advanced imaging (CTP or MRI) if onset was over 6 h or unknown. The stroke team, comprising two neurologists and an interventional neuroradiologist (INR), determined the EVT indication. All patients were examined upon admission and before discharge. Data on patient comorbidities, medications and relevant blood test results, and the modified Thrombolysis in Cerebral Ischemia (mTICI) score [18, 19] were also recorded. All time variables were collected prospectively, and the onset to groin puncture time as well as the procedure time were calculated.

The choice of EVT method was determined by the treating INR. A variety of techniques were employed for clot retrieval, including aspiration with a large-bore catheter and the application of stent retrievers. Permanent stents and percutaneous transluminal angioplasty were utilized when necessary. Immediate post-procedural CT were done in a fraction of patients, while a follow-up MRI or CT scan were done within the first two days. Hemorrhagic complications were labeled using the Heidelberg Bleeding Classification [20]. An increase of ≥ 4 points on the NIHSS secondary to hemorrhagic complications were labeled as symptomatic intracranial hemorrhage (sICH) [21]. The last CT or MRI examination prior to EVT was evaluated retrospectively with regards to ASPECTS by one experienced neuroradiologists blinded to clinical outcomes. When evaluating DWI-ASPECT, only DWI lesions with diameter ≥ 10 mm, visible on at least two adjacent DWI slices in one region, were counted. Three-month follow-ups were conducted through readmission or by telephone interview. Functional outcome was scored using mRS by a mRS certified neurologist, and the population dichotomized to either fair (mRS ≤ 3) or poor (mRS ≥ 4) outcome. The first-year patient mortality status was collected through automatic updates from public records of the OSCAR registry.

### Statistical analyses

All statistical analyses were performed using SPSS version 29 (IBM Corp., Armonk, NY). Bivariate analyses for categorical values were analyzed with the Chi-square test, while continuous variables were compared using the Mann Whitney *U* or Independent *T*-test with respect to their distribution. Logistic regression analyses were used to determine the association between variables in the primary bivariate analyses and to predict functional outcome at the 3-month follow-up. The TERPS for each patient was calculated by summing the transmuted variable values as described in Table 1. Using the calculated TERPS, validation was performed through receiver operating curve (ROC) analysis. A p-value less than 0.05 was considered statistically significant.

## Results

Among the 1028 consecutive patients who received EVT for AIS during the inclusion period, 248 patients (24.2%) were aged ≥ 80 years, of whom 218 (21.2%) patients met the inclusion criteria and were included in the analyses. The median age was 84 (IQR 82–87), and 58.3% were female. Atrial fibrillation was the most common risk factor at 62.8%, followed by hypertension (55%), previous stroke and heart failure (Table 2). The median time from symptom onset or recognition to groin puncture was 250 min (IQR 192–311), while median procedure time was 60 min (IQR 40–85), and puncture to recanalization median was 44 min. Recanalization of mTICI ≥ 2b was achieved in 189 (86.7%) patients. SICH occurred in 4 (1.8%) patients (Table 2). The 1-year survival rate in our cohort was 61.9%.

**Table 2:** Baseline characteristics and group comparison according to functional outcome at 3-month follow-up

Characteristics	*N* = 218	mRS 0–3	mRS 4–6	*p*
Median age, (IQR)	84 (82–87)	84 (82–86)	85 (82–88)	0.025
Sex, female, *n* (%)	127 (58.3)	60 (51.3)	67 (66.3)	0.035
Median pre-stroke mRS, (IQR)	0 (0–2)	0 (0–1)	1 (0–2)	0.002
Baseline NIHSS, median (IQR)	15 (10–19)	12 (8–17)	17 (14–21)	< 0.001
NIHSS at discharge, median (IQR)	8 (3–16)	3(1–6)	16 (16–22)	< 0.001
Intravenous thrombolysis, *n* (%)	114 (52.3)	63 (53.8)	51 (50.5)	0.720
ASPECTS, median (IQR)	8 (6–9)	8 (7–10)	7 (6–9)	0.001
Atrial fibrillation, *n* (%)	137 (62.8)	79 (67.5)	58 (57.4)	0.162
Hypertension, *n* (%)	120 (55.0)	67 (57.3)	53 (52.5)	0.567
Prior stroke/TIA, *n* (%)	58 (26.6)	33 (28.2)	25 (24.8)	0.673
Heart failure, *n* (%)	49 (22.5)	25 (21.4)	24 (23.8)	0.795
Diabetes, *n* (%)	35 (16.1)	15 (12.8)	20 (19.8)	0.224
Antiplatelet medication, *n* (%)	72 (33.0)	39 (33.3)	33 (32.7)	1.00
Anticoagulation, *n* (%)	68 (31.2)	40 (34.2)	28 (27.7)	0.378
Statin treatment, *n* (%)	81 (37.2)	47 (40.2)	34 (33.7)	0.395
Median onset to groin puncture, minutes, (IQR)	250 (192–311)	240 (192–285)	255 (192–327)	0.244
Median groin puncture to end of procedure, minutes (IQR)	60 (40–85)	56 (40–81)	62 (41–91)	0.193
mTICI 2b-3 (%)	189 (86.7)	108 (92.3)	81 (80.2)	0.015
Procedural general anaesthesia, *n* (%)	48 (22.0)	25 (21.4)	23 (22.8)	0.932
Site of occlusion, *n* (%):				0.349
M1	119 (54.6)	58 (49.6)	61 (60.4)	
M2	51 (23.4)	30 (25.6)	21 (20.8)	
M3	5 (2.3)	4 (3.4)	1 (1)	
ICA	9 (4.1)	5 (4.3)	4 (4)	
T occlusion	19 (8.7)	9 (7.7)	10 (9.9)	
Tandem occlusion	15 (6.9)	11 (9.4)	4 (4)	
sICH, *n* (%)	4 (1.8)	0(0)	4(4)	0.045
Hemoglobin, g/dL mean (SD)	12.6 (1.8)	12.9 (1.7)	12.32 (1.9)	0.008
Platelet count, × 10^9^/L, median (IQR)	215 (173–270)	210 (169–265)	222 (178–285)	0.096
INR,, median (IQR)	1.1 (1–1.2)	1.1 (1–1.2)	1.1 (1–1.2)	0.443
Troponin, ng/L, median (IQR)	24 (16–47.5)	24 (16–40)	26 (16–58)	0.159
Creatinine, umol/L, median (IQR)	81 (66.8–102)	89 (71–110)	77 (58–96)	< 0.001
Glucose, mmol/L, median (IQR)	6.9 (6.1–7.9)	6.8 (5.9–7.8)	7.1 (6.4–8.4)	0.020
Low-density lipoprotein, mmol/L, median (IQR)	2.1 (1.6–2.8)	2.2 (1.7–2.8)	2.1 (1.5–2.8)	0.281
C-reactive protein, mg/L, median (IQR)	3.9 (1.4–13)	3.4 (1,.4–10)	5.3 (1.7–15.5)	0.053

Descriptive categorical data were reported as absolute values (percentages), while continuous variables were shown as mean (standard deviation, SD) or median (interquartile range, IQR)

*MRS* modified Rankin scale, *NIHSS* National Institutes of Health Stroke Scale, *ASPECTS* Alberta stroke program early CT score, *mTICI* modified Thrombolysis in Cerebral Infarction, *INR* International normalized ratio, *M1-3* Middle cerebral artery segments 1,2 &3, *ICA* Internal carotid artery

Overall, 117 patients (53.7%) had fair outcome (mRS ≤ 3) at the 3-month follow-up, whereof 95 (81.2%) had TERPS ≤ 7 and 22 (18.8%) with TERPS > 7. The basic characteristics in the group with mRS ≤ 3) and mRS ≥ 4 are shown in Table 2. Male sex, lower age, pre-stroke mRS, NIHSS, as well as higher ASPECT score and recanalization grade were more common in the group with a fair outcome. All patients with sICH had a poor outcome at the 3-month follow-up. Furthermore, higher serum glucose and CRP levels, and lower hemoglobin and creatinine levels were more common in the group with poor outcome.

**Fig. 2:**
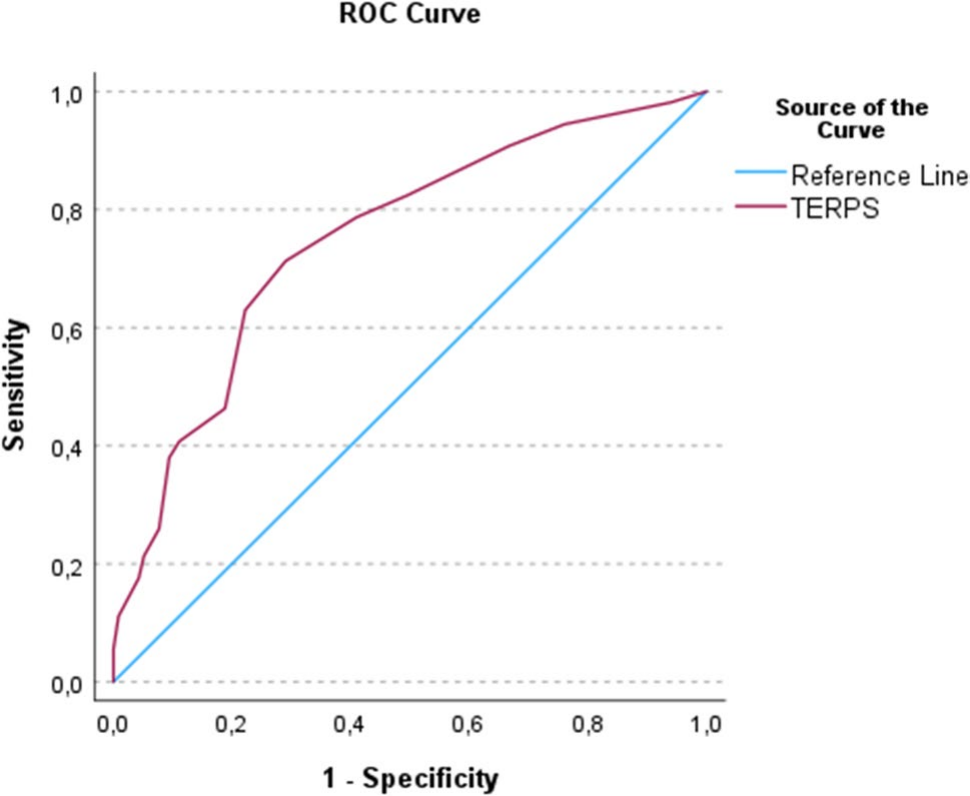
Thrombectomy in the elderly risk prediction score (TERPS) for identification of patients aged ≥ 80 years with fair outcome after thrombectomy using an area under receiver operating curve analysis

**Fig. 3:**
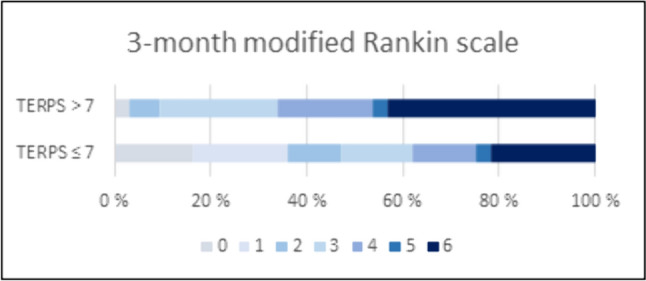
Functional outcome at 3-month follow-up comparing group of patients with Thrombectomy in the elderly risk prediction score (TERPS) ≤ 7 and TERPS > 7

After adjusting for age, gender, hemoglobin, C-reactive protein, and creatinine, multivariate regression analyses showed that lower pre-stroke mRS (adjusted odds ratio [aOR] 0.67; 95% confidence interval [CI]: 0.50–0.91; *p* 0.01), NIHSS (aOR 0.92; 95% CI 0.87–0.97; *p* 0.002), glucose (aOR 0.82; 95% CI 0.68–0.98; *p* 0.030), and higher ASPECTS (aOR 1.22; 95% CI 1.03–1.44; *p* 0.023) were associated with fair outcome. Using 7 as the cut-off value for TERPS, ROC analysis showed an area under the curve value of 0.74 (95% CI 0.67–0.80; *p* < 0.001) (Fig. 2). Out of 153 patients with TERPS ≤ 7, 95 (62%) had fair outcome, while 58 (34%) had poor outcome. In contrast, among the 65 patients with TERPS > 7, 22 (34%) had fair outcome, while 43(66%) had poor outcome. We calculated a sensitivity of 81.2% and specificity of 42.6%. The positive predictive value (PPV) was 62.1%, and the negative predictive value (NPV) was 66.2%. We observed a shift toward a better mRS in patients with TERPS of 0–7 compared to TERPS > 7 (Fig. 3). Testing TERPS for predicting 1-year survival, we calculated an area under the ROC curve of 0.70 (95% CI 0.63–0.77; *p* < 0.001).

## Discussion

In the present study, the clinical risk prediction score TERPS was shown as a potential tool in predicting fair functional outcome in patients ≥ 80 years treated with EVT. The ROC analysis showed an AUC of 0.74 (95% CI 0.67–0.80; *p* < 0.001) for 3-month functional level and an AUC of 0.70 (95% CI 0.63–0.77; *p* < 0.001) for 1-year survival.

Compared to the Danish cohort, from which the TERPS was derived and tested, we found comparable sensitivity (0.81 vs 0.86) and PPV (0.62 vs 0.57), while specificity (0.42 vs 0.52) and NPV (0.66 vs 0.84) were lower in our cohort. Laugesen et.al. noted that poor outcome occurred with similar prevalence above and below the cut-off value [17]. Elderly with a TERPS ≤ 7 are good candidates for EVT and should be treated given that no other contraindications exist. The cohort that the present study was based on is comparable to the cohort that TERPS was originally developed from. Both cohorts included all patients receiving EVT in both anterior and posterior circulation, including both LVO and MeVO [17].

Various prediction scores have been published in the field, including MR PREDICTS, a browser-based prediction model incorporating 12 variables for all age groups [22]. In contrast, TERPS, comprising only four variables, offers simpler usability. It is also developed specifically for elderly patients and had almost three times as many patients with a pre-stroke mRS of 3 than MR PREDICTS derivation cohort (11% vs 4%) [17, 23]. Advancing age is widely recognized to be associated to worse outcomes following stroke and EVT. A recent systematic review and meta-analysis reports that the effect of EVT is more robust in younger patients [24]. The ETIS Registry study conducted by Finitsis et al. found a robust correlation between age and 3-month mRS [25], a finding congruent with the research by Beuker et al. who also demonstrated a similar link between age and 1-year survival post-EVT [26]. These represent just a couple of instances among several studies that have included age as a stratification factor in their analyses. While we found NIHSS, pre-stroke mRS, ASPECTS, and blood glucose as predictors of outcomes, it was surprising that age did not emerge as a significant predictor within our dataset. One possible explanation for this observation could be that, within our elderly population, the impact of age diminished when individuals underwent stricter selection process with advancing age, potentially influencing the results. Including all age groups in predicting models can possibly affect estimated outcome for the elderly in less stringent selection for EVT.

The pre-stroke mRS has become widely utilized in both clinical practice and research. One of its earlier applications in discerning patient’s premorbid status was conducted by McNaughton et.al. [27], where they found an association between Barthel Index rate of change and pre-stroke mRS. Similarly, Foell et.al. used pre-stroke mRS in scoring patients with disabilities receiving thrombolysis [28]. Debates have arisen regarding its use in assessing premorbid function [16, 29]. Quinn et al. reported the fair validity of pre-stroke mRS, noting a moderate correlation with other measures reflecting pre-stroke disability [30], while Zhang et.al. found emergency department-assessed pre-stroke mRS to be reliable, after comparing scores obtained from detailed functional information acquired thereafter [31]. The practicality of using pre-stroke mRS and 3-month mRS lies in its simplicity and ease of comparison. Even though EVT is more effective compared to medical management, even in patients with increasing pre-stroke mRS, an increase in pre-stroke mRS has been linked with worse prognosis or death [2, 12, 32]. Additionally, the elderly with pre-stroke disability may have a further increased risk of worse outcome [33], as exemplified in this study.

Association between hyperglycemia and unfavorable outcome, sICH and development of malignant edema during and after EVT has been reported in previous studies [34, 35]. In the MR PREDICTS score, the glucose variable was added, improving their model [22]. Thus, glucose levels can be a candidate 5th variable that may enhance the model yet keep it simple for clinical practice.

Aside from blood glucose, lower hemoglobin and creatinine were associated with poor outcome on initial bivariate analysis. Although these factors are not routinely used in deciding treatment, they do reflect the patient’s current health state. There is evidence that anemia and frailty have an entangled association and may predispose to poor outcome in sick elderly [36]. Creatinine, a byproduct of creatine, also reflects the lean muscle mass of an individual. A very recent study has shown that creatinine, calculating a derived creatinine muscle index, is associated with frailty and increased mortality [37]. As more studies reports an association between frailty and stroke outcome [38], a consensus among stroke physicians on which frailty scale to use in the acute setting is still lacking.

An important limitation of our study is that TERPS was evaluated within a cohort comprising exclusively of patients already preselected for thrombectomy. Consequently, cautious consideration is warranted when interpreting the score in clinical practice as a means of excluding patients from EVT. This caution is underscored by the notably low specificity and NPV observed in our study, as TERPS cutoff over 7 was unable to effectively identify patients with unfavorable outcomes following treatment.

## Conclusion

The TERPS could be a practical clinical tool which can guide clinicians in selecting the elderly who may benefit from EVT. Those having scores between 0 and 7 are good candidates for treatment. The association between hyperglycemia and functional outcome can be further investigated as a candidate variable to improve the prediction model. The TERPS showed to be of limited value for predicting poor outcomes and should not be solely used in rejecting patients for EVT in its present form.

## Data Availability

Data gathered and used in this study are available upon reasonable request from the corresponding author.
